# Lifestyle and Follow-Up Gaps Contribute to Poor Glycemic Control in Patients With Type 2 Diabetes Mellitus in Southwestern Bangladesh

**DOI:** 10.7759/cureus.94463

**Published:** 2025-10-13

**Authors:** Kishore Kumar Shil, Sudipta Bakchi, Susanta K Paul, Mahmud Hossain

**Affiliations:** 1 Endocrinology, Khulna Medical College, Khulna, BGD; 2 Cardiology, Khulna Medical College Hospital, Khulna, BGD; 3 Pulmonology, Khulna Medical College Hospital, Khulna, BGD; 4 Rheumatology, Khulna Medical College Hospital, Khulna, BGD

**Keywords:** lifestyle intervention, long-term follow-up, poor glycemic control, risk predictors, type 2 diabetes

## Abstract

​​​Background

Type 2 diabetes mellitus (T2DM) is a growing global health concern, particularly in low- and middle-income countries, where the rate of poor glycemic control is high. Lifestyle modifications and regular follow-ups are essential for effective management and prevention of complications.

Methodology

This cross-sectional study was conducted at a tertiary care facility from March to December 2024, enrolling 360 adults with T2DM using non-probability purposive sampling. Data on demographics, clinical history, and biochemical parameters (glycated hemoglobin (HbA1c), plasma glucose) were collected to assess glycemic control, with an HbA1c threshold of 7%. Ethical approval and informed consent were obtained, and data were analyzed using SPSS version 25 (IBM Corp., Armonk, NY, USA).

Results

A total of 360 adults with T2DM participated in the study, with a mean age of 48.4 years. Most participants were female (62.7%) and overweight or obese (78%). The median duration of diabetes was five years, and 60% of patients had at least one comorbidity. Poor glycemic control was found in 82.7% based on HbA1c and 90.2% based on plasma glucose. Better glycemic control was significantly associated with factors such as shorter diabetes duration, medication adherence, diabetes education, diet, exercise, and regular follow-up. Logistic regression revealed that age, diabetes duration, diabetic diet (odds ratio (OR) = 5.6), regular exercise (OR = 3.8), and regular follow-up (OR = 8.5) were significantly associated with better glycemic control.

Conclusions

The study revealed a high prevalence of poor glycemic control among T2DM patients in southwestern Bangladesh. Targeted interventions, especially lifestyle and regular follow-up protocols, are urgently needed to improve diabetes management and prevent complications.

## Introduction

Diabetes mellitus (DM) is a chronic metabolic disorder characterized by elevated blood glucose levels resulting from insulin deficiency or resistance [1]. Type 2 diabetes mellitus (T2DM) accounts for approximately 90% of all DM cases. If left uncontrolled, persistent hyperglycemia can lead to complications involving the eyes, kidneys, nerves, heart, and blood vessels [2]. Globally, DM affects over 422 million adults and is projected to reach 642 million by 2040, contributing to nearly 5 million deaths each year [3,4]. Approximately 80% of diabetes cases occur in low- and middle-income countries (LMICs), where limited healthcare resources and access contribute to poorer outcomes [5].

Bangladesh ranks among the top 10 LMICs most affected by diabetes, with the number of cases expected to rise from 8.4 million in 2019 to 15 million by 2045 [6]. The national prevalence is estimated at 12.5%, with higher rates in urban areas [7]. According to the 2017-2018 Bangladesh Demographic and Health Survey, only 26% of individuals with diabetes had their blood glucose levels under control, 31% were aware of their diagnosis, and 28% were receiving treatment [8]. Other studies have also reported suboptimal glycemic control and a high burden of complications among patients with T2DM in Bangladesh [9-11].

Multiple barriers contribute to poor glycemic control in the region. Patient-related factors include limited knowledge, poor medication adherence, and difficulties in dietary regulation. Additionally, systemic barriers such as inadequate healthcare infrastructure and limited access to specialists complicate diabetes management [12]. Cultural habits, including a rice-dominant diet and low levels of physical activity, further hinder effective glycemic control [13,14].

Identifying predictors of glycemic control is crucial for reducing diabetes-related complications and improving clinical outcomes [11]. Despite the availability of evidence-based guidelines, many patients struggle to achieve target glycemic levels due to the complex interplay of individual, cultural, and systemic factors. This study aims to assess the patterns and predictors of glycemic control among patients with T2DM in southwestern Bangladesh, with the goal of informing targeted, context-specific interventions.

## Materials and methods

This study employed a cross-sectional analytical design conducted at a tertiary care hospital between March 2024 and December 2024. Participants were recruited from the outpatient department and through referrals using a non-probability purposive sampling, until the target sample size was achieved. Eligible participants included male and female adults aged 18 years or older, diagnosed with T2DM, who attended the diabetes clinic and provided written informed consent. Exclusion criteria included critically ill patients and individuals who declined to sign the consent form.

Data were collected by trained research assistants who received standardized training in study protocols. All measurements and interviews were conducted under the supervision of the principal investigator to ensure accuracy and consistency. Data were gathered using a pre-tested, structured questionnaire that included sections on demographic, clinical, lifestyle, and treatment-related factors. The questionnaire was developed based on previously validated tools and adapted to the local context after pilot testing. Minor modifications were made for cultural appropriateness, and internal consistency was assessed before data collection.

Data collection encompassed demographic information (residence, name, age, sex, monthly income), diabetes history (duration of diabetes, prior antidiabetic treatments, exercise habits, dietary practices, diabetes education, and regular follow-up), clinical parameters (height, weight, body mass index (BMI), and blood pressure), and biochemical measures (fasting plasma glucose, two-hour plasma glucose, and glycated hemoglobin (HbA1c)). Biochemical tests were conducted at accredited laboratories during participants’ routine follow-up care. Height was measured using a stadiometer, while weight was measured on a calibrated balance placed on a hard, flat surface. Blood pressure was measured in millimeters of mercury using a standard sphygmomanometer. Hypertension was defined as having a documented hypertension diagnosis, current use of antihypertensive medications, or recent (within three months) blood pressure readings with systolic ≥140 mmHg or diastolic ≥90 mmHg.

BMI was categorized according to the World Health Organization (WHO) adult obesity classification for Asian populations: underweight, <18.5 kg/m²; normal weight, 18.5-22.9 kg/m²; overweight, 23-24.9 kg/m²; and obese, ≥25 kg/m². Glycemic control was assessed using HbA1c, with levels <7% considered good control and ≥7% considered poor control.

Diabetes education was categorized into the following three levels: (1) no education: individuals who had never received any form of diabetes-related education; (2) unstructured education: individuals who had received informal advice from peers, family members, or neighbors with diabetes, but not from certified healthcare professionals; and (3) structured education: individuals who had received formal, organized education sessions provided by certified diabetes educators or diabetes specialists, focusing on disease knowledge, self-management, and lifestyle modification.

Diet adherence was assessed based on individualized dietary recommendations tailored to patients’ comorbidities and affordability. The standard recommendations emphasized a meal pattern of five to six times daily (three main meals and two to three snacks); appropriate portion control; reduction of refined carbohydrates, fats, sugars, and processed foods; and increased intake of vegetables and non-starchy carbohydrates.

Dietary adherence was categorized into the following three levels: (1) full adherence: reported daily compliance with recommended dietary guidelines for diabetes, including controlled portion size, reduced carbohydrate and fat intake, and regular meal timing; (2) partial adherence: reported following dietary recommendations inconsistently (e.g., ≥3 days per week) or with occasional lapses in portion control and food choice; and (3) non-adherence: rare or no compliance with dietary recommendations, including unrestricted intake of sweets, fried foods, or irregular meal timing.

Medication adherence was categorized into the following using a combination of self-report and pill count data: (1) good adherence: rarely missed doses (≤2 missed doses per month) and regular refills/pill count concordance; (2) partial adherence: occasionally missed doses (>2 but <8 missed doses per month) or irregular refills; and (3) non-adherence: frequently missed doses (≥8 per month) or reported discontinuation without medical advice.

Exercise adherence was classified into the following based on reported physical activity relative to WHO recommendations: (1) adherent: engaged in ≥150 minutes of moderate-intensity aerobic activity per week (e.g., brisk walking, cycling) or ≥75 minutes of vigorous-intensity activity, spread across ≥3 days; (2) partial adherence: reported some activity (<150 minutes per week or not maintain intensity) but less than the recommended threshold; and (3) non-adherence: reported no regular physical activity.

Sample size was calculated using the standard formula described by Kish Leslie, considering prevalence for a single population: n =  ­Z² × p (100−p)/e227, assuming standard normal variables (z score) of 1.96 at 95% confidence interval, margin error (e) of 5% and prevalence (p) of 75.8% of poor glycemic control. The total sample size obtained was 360 after considering an attrition rate of 10%.

Statistical analysis

Statistical analysis was performed using SPSS Statistics for Windows, version 25.0 (IBM Corp., Armonk, NY, USA). Quantitative data were expressed as mean ± standard deviation (SD) or median with interquartile range (IQR), while categorical data were presented as frequencies and percentages. Associations between categorical variables were analyzed using the chi-square test or Fisher’s exact test, as appropriate. Multiple logistic regression analysis was used to identify predictors of glycemic control. Predictors were selected based on clinical relevance and prior evidence. Multicollinearity was assessed using variance inflation factor values, all of which were close to 1 (1.04-1.49), indicating no significant collinearity. Model fit was evaluated using the Hosmer-Lemeshow test (p = 0.079), omnibus test (p < 0.001), and the C-statistic (area under the curve = 0.88), demonstrating good calibration and excellent discrimination. The events-per-variable ratio was 1.5 for the controlled DM outcome and 7.5 for the uncontrolled DM outcome, which may limit model stability; therefore, alternative approaches such as penalized regression could be considered in future studies. A P-value <0.05 was considered statistically significant for all tests.

Ethical considerations

Informed written consent was obtained from all participants after providing detailed information on the study’s purpose and procedures. Participants retained the right to participate, refuse, or withdraw at any point without affecting their access to medical care. All participants received appropriate medical services and counseling regardless of study participation status. Confidentiality of patient information was strictly maintained. The study protocol was approved by the Ethical Review Committee of Khulna Medical College Hospital (reference number: KMC/ERC/03; dated 05 March 2024), Khulna, Bangladesh. All procedures complied with the ethical principles outlined in the Declaration of Helsinki.

## Results

A total of 360 T2DM patients were included in this study to assess glycemic control and its predictors. Participants had a mean age of 48.4 ± 11.4 years (range = 18-86 years), with 62.7% aged 41-60 years. Most were female (62.7%) and overweight/obese (78%) with a mean BMI of 25.6 ± 4.2 kg/m². The median diabetes duration was five years. Comorbidities were reported in 60%, and 55% regularly used antidiabetic medication. However, 64.4% missed regular three-month follow-ups, only 10.8% followed a diabetic diet, 16.6% exercised regularly, and a similar percentage received structured diabetes education (Table 1).

**Table 1:** Demographic and clinical characteristics of the study participants (N = 360). Mean ± SD for normally distributed data and median (IQR) for skewed data. Within parentheses are percentages over the column total, if not mentioned otherwise. DM: diabetes mellitus; BP: blood pressure; BMI: body mass index; HTN: hypertension; DL: dyslipidemia; CKD: chronic kidney disease; IQR: interquartile range

Variables	n (%)
Age (years); mean ±SD	48.4 ± 11.4
Age category (years)	
18–40	101 (28.0)
41–60	208 (57.7)
≥61	51 (14.1)
Gender	
Male	134 (37.2)
Female	226 (62.7)
DM duration (years; median and IQR)	5.0 (3.0-6.0)
DM duration category (years)	
<5	145 (40.2)
5–10	144 (40.0)
≥11	71 (19.7)
Income category (tk)	
<20,000	224 (62.2)
21,000–40,000	125 (34.7)
41,000–60,000	11 (3.0)
BMI (kg/m^2^)	25.6 ± 4.2
BMI category	
Normal	79 (21.9)
Overweight/Obese	281 (78.0)
Residence	
Rural	186 (51.6)
Urban	174 (48.3)
Comorbidities	
Yes	216 (60%)
No	144 (40%)
Types of comorbidities	
HTN	88 (40.7%)
DL	65 (30.0%)
HTN + DL	37 (17.1%)
Hypothyroidism	19 (8.7%)
CKD	7 (3.2%)
Regular intake of antidiabetic medication	
No	162 (45.0)
Yes	198 (55.0)
Diabetic education	
No	125 (34.7)
Unstructured	197 (54.7)
Diabetic diet	
No	139 (38.6)
Partial	182 (50.5)
Yes	39 (10.8)
Exercise	
No	169 (46.9)
Partial	131 (36.3)
Yes	60 (16.6)
Follow-up (≤3 monthly)	
No	232 (64.4)
Yes	128 (35.5)

Biochemical results showed elevated glycemic levels. The mean fasting plasma glucose was 11.2 ± 4.2 mmol/L, two-hour plasma glucose was 16.2 ± 5.7 mmol/L, and HbA1c was 9.3 ± 2.3% (Table 2).

**Table 2:** Glycemic values of the study participants (N = 360). FPG: fasting plasma glucose; 2h-PG: two-hour plasma glucose; HbA1c: glycated hemoglobin

Variable	Values (mean ±SD)
FPG (mmol/L)	11.2 ± 4.2
2h-PG (mmol/L)	16.2 ± 5.7
HbA1c (%)	9.3 ± 2.3

**Figure 1:**
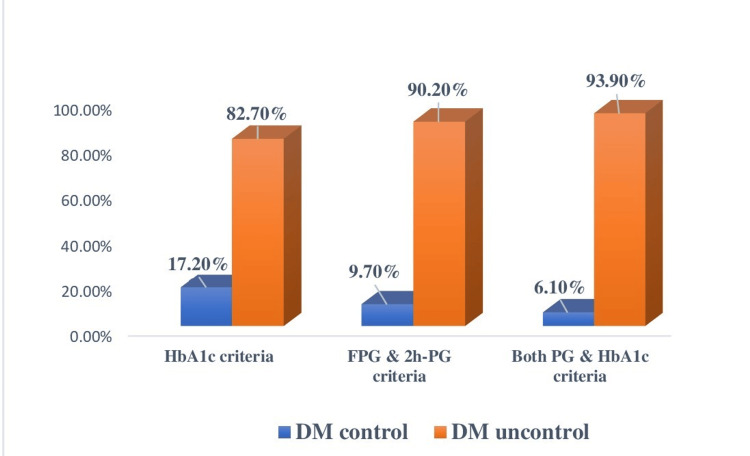
Glycemic status of the study participants (n = 360). DM control: HbA1c <7%, FPG = 4.7-7.2 mmol/L, and 2h-PG <10 mmol/L. DM: diabetes mellitus; FPG: fasting plasma glucose; 2h-PG: two-hour plasma glucose; HbA1c: glycated hemoglobin

Poor glycemic control was found in 82.7% by HbA1c, 90.2% by plasma glucose, and 93.9% by both criteria (Figure 1).

There was no significant association of glycemic control (HbA1c <7%) with age, gender, residence, income, or BMI. However, diabetes duration, regular medication, diabetes education, diet adherence, exercise, and regular follow-up were significantly associated with better control. Those on regular medication had higher control rates (70.9% vs. 51.6%), as did participants with structured (25.8% vs. 7.3%) or unstructured (61.2% vs. 48.3%) education. Exercise adherence and regular follow-ups also improved control (56.4% vs. 32.2% and 83.8% vs. 25.5%, respectively) (Table 3).

**Table 3:** Comparison of characteristics of participants according to glycemic control. Significance level was measured by c2 test; P<0.05 is significant. DM: diabetes mellitus; BMI: body mass index; BP: blood pressure; DM controlled: HbA1c <7 %; DM uncontrolled: HbA1c ≥7%

Characteristics	Controlled DM (n = 62)	Uncontrolled DM (n = 298)	P-value	Chi-square value
Age group (years); n (%)			0.23	2.93
18–40	12 (19.3)	89 (29.8)		
41–60	41 (66.1)	167 (56.0)		
≥61	9 (14.5)	42 (14.0)		
Gender; n (%)			0.15	2.02
Male	28 (45.1)	106 (35.5)		
Female	34 (54.8)	192 (64.4)		
Residence; n (%)			0.40	0.68
Rural	35 (56.4)	151 (50.6)		
Urban	27 (43.5)	147 (49.3)		
Income category (tk.); n (%)			0.66	0.80
<20,000	38 (61.2)	186 (62.4)		
21,000–40,000	21 (33.8)	104 (34.8)		
41,000–60,000	3 (4.8)	8 (2.68)		
DM duration (years); n (%)			0.01	8.14
<5	35 (56.4)	110 (36.9)		
5–10	18 (29.0)	126 (42.2)		
≥11	9 (14.5)	62 (20.8)		
Regular intake of antidiabetic medication; n (%)			0.003	9.07
No	18 (29.0)	144 (48.3)		
Yes	44 (70.96)	154 (51.6)		
DM education; n (%)			<0.001	27.36
No	8 (12.9)	117 (39.2)		
Unstructured	38 (61.2)	159 (53.3)		
Structured	16 (25.8)	22 (7.3)		
Diabetic diet; n (%)			<0.001	40.84
No	6 (9.6)	133 (44.6)		
Partial	38 (61.2)	144 (48.3)		
Yes	18 (29.0)	21 (7.0)		
Exercise; n (%)			<0.001	35.55
No	8 (12.9)	161 (54.0)		
Partial	35 (56.4)	96 (32.2)		
Yes	19 (30.6)	41 (13.7)		
Follow-up; n (%)			<0.001	76.30
No	10 (16.1)	222 (74.4)		
Yes	52 (83.8)	76 (25.5)		
BMI category (kg/m^2)^;n (%)			0.58	0.29
Normal	12 (19.3)	67 (22.4)		
Overweight/obese	50 (80.6)	231 (77.5)		
BP category; n (%)			0.08	3.04
Normal	36 (58.0)	207 (69.4)		
High	26 (41.9)	91 (30.5)		

Multiple regression analysis was performed to determine the predictors of glycemic control (HbA1c ≥7%) (Table 4). Multiple logistic regression showed that gender, BMI, income, and diabetic education were not significantly associated with good glycemic control. Participants on regular antidiabetic medication were 1.26 times more likely (95% confidence interval (CI) = 0.56-2.82), and those with structured education were 2.17 times more likely (95% CI = 0.61-7.68) to achieve good control. Key predictors of good glycemic control included age, regular follow-up (≤3 months), exercise, dietary adherence, and diabetes duration. Participants aged 41-60 were 3.6 times more likely to achieve good control than those ≤40 years (p = 0.006). Those with diabetes for 5-10 years were 0.3 times less likely to maintain good control than those diagnosed ≤5 years (p = 0.03). Moreover, adherence to a diabetic diet (odds ratio (OR) = 5.6, 95% CI = 1.4-22.5, p = 0.01), regular exercise (OR = 3.8, 95% CI = 1.3-10.8, p = 0.01), and regular follow-up (OR = 8.5, 95% CI = 3.6-20.1, p < 0.001) were strongly associated with good glycemic control.

**Table 4:** Multiple logistic regression for predictors of good glycemic control. R² = 27.8-46.3 %; p < 0.001. P-values <0.05 are significant. Glycemic control: HbA1c <7%. OR: odds ratio; CI: confidence interval; BMI: body mass index; HbA1c: glycated hemoglobin

Variables	B	OR	P-value	95% CI	
				Lower	Upper
Age category (years); (reference: 18–40)	-	-	0.02	-	-
41–60	1.28	3.62	0.006	1.4	9.0
≥61	1.01	2.75	0.10	0.8	9.2
Sex (reference: male)	-0.38	0.68	0.31	0.32	1.44
BMI (kg/m^2^)	0.006	1.00	0.91	0.90	1.12
Income: tk. (reference: < 20000)	-	-	0.61	-	-
20,000–40,000	0.33	1.39	0.42	0.62	3.13
≥41,000	0.66	1.94	0.46	0.33	11.4
Residence (reference: rural)	-0.20	0.98	0.95	0.47	2.01
Duration of DM (<5 years)	-	-	0.06	-	-
5–10	-1.17	0.30	0.03	0.10	0.90
≥11	-1.07	0.34	0.37	0.03	3.72
Regular intake of antidiabetic medication (reference: no)	0.23	1.26	0.57	0.56	2.82
Diabetes education (reference: no)	-	-	0.20	-	-
Unstructured	-0.90	0.91	0.86	0.33	2.53
Structured	0.77	2.17	0.22	0.61	7.68
Diabetics diet (reference: no)	-	-	0.04	-	-
Partial	1.05	2.85	0.06	0.95	8.53
Yes	1.72	5.62	0.01	1.41	22.45
Exercise (reference: no)	-	-	0.04	-	-
Partial	0.84	2.33	0.13	0.76	7.14
Yes	1.32	3.75	0.01	1.30	10.75
Follow-up (≤3 monthly) (reference: no)	2.14	8.52	0.001	3.61	20.10

## Discussion

Effective glycemic control remains a cornerstone in the management of T2DM, as it significantly reduces the risk of both microvascular complications, such as retinopathy, nephropathy, and neuropathy, and macrovascular complications, such as cardiovascular disease and stroke. This study found that a substantial majority (82.7%) of participants exhibited poor glycemic control based on HbA1c criteria, indicating a serious gap in diabetes management in southwestern Bangladesh. These findings are consistent with prior studies in similar settings that report widespread challenges in achieving glycemic targets among patients with T2DM [15].

The influence of diabetes duration on glycemic control observed in this study echoes previous research showing that longer disease duration correlates with progressive pancreatic β-cell dysfunction, increased insulin resistance, and often greater challenges in maintaining lifestyle modifications [16,17]. Patients with longer disease duration may also require more complex treatment regimens, including insulin therapy, which can complicate adherence. The lower glycemic control rates observed in these patients highlight the need for intensified clinical support and education as diabetes progresses.

Interestingly, unlike many studies from other LMICs, this study found no significant differences in glycemic control by gender, rural versus urban residence, BMI, or income level [15,18]. This may reflect relatively equitable access to diabetes care in Bangladesh, where government programs provide subsidized medications and standardized treatment protocols that mitigate socioeconomic and geographic disparities. However, further research is needed to confirm whether this pattern holds in larger, nationally representative samples.

Medication adherence was another key determinant of glycemic control, reinforcing its central role in diabetes management. Medication adherence, although significant in the bivariate analysis, lost significance in the multivariate model. This likely reflects confounding by other variables such as follow-up, diet, and exercise, which may account for much of the variation initially attributed to adherence. Participants regularly taking prescribed antidiabetic medications were more likely to achieve glycemic targets, consistent with findings from prior studies emphasizing the importance of compliance to reduce morbidity and mortality in T2DM patients [19].

The roles of diet and physical activity as modifiable factors cannot be overstated. Lifestyle interventions are fundamental to diabetes management and can reduce reliance on pharmacotherapy when adhered to consistently [20,21]. Our findings support these established clinical recommendations, demonstrating that patients adhering to a diabetic diet or engaging in regular exercise had significantly better glycemic control.

Despite the majority of participants being prescribed antidiabetic medications, only a small proportion adhered to key lifestyle recommendations, i.e., approximately 10% followed a diabetic diet, 17% engaged in regular exercise, and a similar proportion participated in diabetes education programs. This low uptake of lifestyle modification is concerning, given the well-established benefits of diet and physical activity in improving insulin sensitivity and glycemic control [20,21]. The low participation in diabetes education programs suggests gaps in patient awareness and healthcare delivery, underscoring an urgent need for more accessible, culturally tailored educational interventions.

Our analysis identified several significant predictors of better glycemic control, including regular follow-up visits (at least every three months), adherence to a diabetic diet, regular exercise, shorter duration of diabetes, and age between 41 and 60 years. These results align well with existing literature demonstrating that patients who actively engage with healthcare providers through regular visits tend to have improved medication adherence, self-care behaviors, and clinical outcomes [21-23]. The strong association of regular follow-up with good glycemic control (OR = 8.5) highlights the critical importance of healthcare system factors in chronic disease management.

The cross-sectional design of this study limits our ability to draw causal conclusions or evaluate temporal changes in glycemic control and its predictors. This study could not account for all potential confounders due to time and resource constraints, such as medication regimens, mental health status, and detailed socioeconomic factors, which may affect interpretation. Additionally, findings are limited to patients at tertiary care facilities and may not be generalizable to the broader diabetes population. Potential inaccuracies arising from self-reported data, including medication adherence and lifestyle factors, and variability in laboratory measurements, despite using accredited labs. However, the strong associations observed provide valuable insights for public health initiatives. The study’s inclusion of multiple modifiable factors, i.e., medication adherence, lifestyle behaviors, and healthcare engagement, alongside non-modifiable factors such as age and disease duration, strengthens its practical relevance for intervention design.

## Conclusions

This study revealed a high prevalence of poor glycemic control among T2DM patients in southwestern Bangladesh, with over 80% not meeting HbA1c targets. Key predictors of good control included a healthy diet, physical activity, and regular follow-ups. Despite over half of the participants adhering to medications, inadequate lifestyle practices and follow-up limited effective control. Although these findings may not represent the broader diabetes population, they highlight the urgent need for integrated, patient-centered interventions to improve diabetes management and reduce complications. Despite the cross-sectional design limiting causal inference, the study offers valuable insights to guide future research and healthcare strategies in resource-limited settings. Future studies should employ probability-based sampling across multiple healthcare settings, including primary care facilities and community settings.
